# Dimethyl 2,2′-(*p*-phenyl­enedi­oxy)­diacetate

**DOI:** 10.1107/S1600536809002980

**Published:** 2009-01-28

**Authors:** Ling-hua Zhuang, Guo-wei Wang

**Affiliations:** a Department of Applied Chemistry, College of Science, Nanjing University of Technology, Nanjing 210009, People’s Republic of China; b Department of Light Chemical Engineering, College of Science, Nanjing University of Technology, Nanjing 210009, People’s Republic of China

## Abstract

The title compound, C_12_H_14_O_6_, was prepared by the Williamson reaction of 1,4-dihydroxy­benzene and methyl chloro­acetate with phase-transfer catalysis. The compound lies on an inversion center. The structure is stabilized by weak C—H⋯π inter­actions.

## Related literature

For details of the synthesis procedure and the applications of benzothia­zoles, see: Chakraborti *et al.* (2004 ▶); Seijas *et al.* (2007 ▶); Wang *et al.* (2009 ▶). For details of the synthesis procedure and the applications of aryl­oxyacetic acids, see: Nagy *et al.* (1997 ▶); Wei *et al.* (2005 ▶). For the use of phase-transfer catalysis in organic synthesis, see: Perreux *et al.* (2001 ▶). For bond-length data, see: Allen *et al.* (1987 ▶).
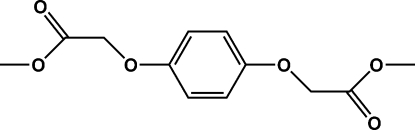


## Experimental

### 

#### Crystal data

C_12_H_14_O_6_
*M* *_r_* = 254.23
Monoclinic, 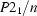

*a* = 7.4190 (15) Å
*b* = 7.0990 (14) Å
*c* = 11.785 (2) Å
β = 95.49 (3)°
*V* = 617.8 (2) Å^3^
*Z* = 2
Mo *K*α radiation
μ = 0.11 mm^−1^
*T* = 293 (2) K
0.30 × 0.20 × 0.10 mm

#### Data collection

Enraf–Nonius CAD-4 diffractometer
Absorption correction: ψ scan (North *et al.*, 1968 ▶) *T*_min_ = 0.954, *T*_max_ = 0.977
1123 measured reflections
1123 independent reflections
769 reflections with *I* > 2σ(*I*)
3 standard reflections every 200 reflections intensity decay: 9%

#### Refinement

*R*[*F* ^2^ > 2σ(*F* ^2^)] = 0.058
*wR*(*F* ^2^) = 0.173
*S* = 1.00
1123 reflections
82 parameters
H-atom parameters constrained
Δρ_max_ = 0.26 e Å^−3^
Δρ_min_ = −0.24 e Å^−3^

### 

Data collection: *CAD-4 Software* (Enraf–Nonius, 1989 ▶); cell refinement: *CAD-4 Software*; data reduction: *XCAD4* (Harms & Wocadlo, 1995 ▶); program(s) used to solve structure: *SHELXS97* (Sheldrick, 2008 ▶); program(s) used to refine structure: *SHELXL97* (Sheldrick, 2008 ▶); molecular graphics: *SHELXTL* (Sheldrick, 2008 ▶); software used to prepare material for publication: *SHELXTL*.

## Supplementary Material

Crystal structure: contains datablocks global, I. DOI: 10.1107/S1600536809002980/dn2426sup1.cif

Structure factors: contains datablocks I. DOI: 10.1107/S1600536809002980/dn2426Isup2.hkl

Additional supplementary materials:  crystallographic information; 3D view; checkCIF report

## Figures and Tables

**Table 1:** Hydrogen-bond geometry (Å, °) *Cg*1 is the centroid of the C1–C3/C1*A*–C3*A* ring.

*D*—H⋯*A*	*D*—H	H⋯*A*	*D*⋯*A*	*D*—H⋯*A*
C6—H6*C*⋯*Cg*1^i^	0.97	2.98	3.674 (2)	130

Symmetry code: (i) 
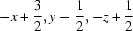
.

**Fig. 1.**
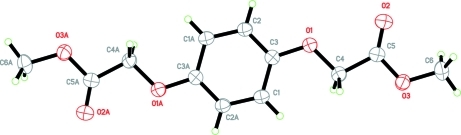
A view of the molecular structure of (I) showing the atom-numbering scheme. Ellipsoids are drawn at the 30% probability level. H atoms are represented as small spheres of arbitrary radii. [symmetry code: (A) 1/2-x, 1/2+y, 1/2-z].

## supplementary crystallographic information

### Comment

The derivatives of aryloxyacetic acids and their derivatives constitute a class of compounds for both biological activity and plant growth regulators (Nagy *et al.*, 1997; Wei *et al.*,2005). The phase-transfer catalysis, with the advantages of simple experimental operations, mild reaction conditions, and inexpensive and environmentally benign reagents, has established its significance in organic synthesis as one of the most useful methods for the acceleration of heterogeneous reactions (Perreux & Loupy, 2001).

Benzothiazole are remarkable heterocyclic ring systems. They have been found to exhibit a wide spectrum of biological activities. Many kinds of 2-substituted benzothiazoles are utilized as vulcanization accelators in the manufacture of rubber,as fluorescent brightening agents in textile dyeing,and in the leather industry (Chakraborti *et al.*,2004; Seijas *et al.*,2007; Wang *et al.*,2009). There are numerous synthetic methods to produce 2-arylbenzothiazoles. The most important ones include the reaction of *o*-aminothiophenols with benzoic acids or their derivatives (Chakraborti *et al.*,2004; Seijas *et al.*,2007; Wang *et al.*,2009). We are focusing on the synthesis of new products of bisbenzothiazole. We here report the crystal structure of the title compound (I).

The compound (I) lies on an inversion center(Fig.1). All bond lengths are within normal ranges (Allen *et al.*, 1987). There are no typical hydrogen bonds, while weak intermolecular C—H···π interactions involving benzene ring (C1/C3/C2/C1a/C3a/C2a) (Table 1) may help in stabilizing the structure.

### Experimental

5.5 g (0.05 mole) hydroquinone was dissolved in 50 ml acetone, 6.9 g (0.05 mole) potassium carbonate, potassium iodide 0.8 g and tetrabutyl ammonium bromide 1.0 g were added. Then 8.8 ml L (0.10 mole) of methyl chloroacetate was dropped into the mixture. The mixture was boiled for 5 h with intensive stirring, cooled to room temperature, and filtered. The organic solution was evaporated under vacuum to dryness and the dry residue was recrystallized from methanol to obtain title compound. Crystals of (I) suitable for X-ray diffraction were obtained by slow evaporation of ethyl acetate. ^1^H NMR (CDCl_3_, δ, p.p.m.) 6.85 (m, 4H), 4.58 (s, 4H), 3.79 (s,6*H*).

### Refinement

All H atoms were positioned geometrically and treated as riding on their parent C atoms with C—H = 0.93 Å (aromatic), 0.97Å (methylene) and 0.96Å (methyl) with *U*_iso_(H) = *xU*_eq_(C), where *x*= 1.5 for methyl H and 1.2 for aromatic and methylene H atoms.

### Crystal data

**Table:**

C_12_H_14_O_6_	*F*(000) = 268
*M**_r_* = 254.23	*D*_x_ = 1.367 Mg m^−^^3^
Monoclinic, *P*2_1_/*n*	Mo *K*α radiation, λ = 0.71073 Å
Hall symbol: -P 2yn	Cell parameters from 27 reflections
*a* = 7.4190 (15) Å	θ = 1–25°
*b* = 7.0990 (14) Å	µ = 0.11 mm^−^^1^
*c* = 11.785 (2) Å	*T* = 293 K
β = 95.49 (3)°	Block, yellow
*V* = 617.8 (2) Å^3^	0.30 × 0.20 × 0.10 mm
*Z* = 2	

### Data collection

**Table:**

Enraf–Nonius CAD-4 diffractometer	769 reflections with *I* > 2σ(*I*)
Radiation source: fine-focus sealed tube	*R*_int_ = 0.0000
graphite	θ_max_ = 25.3°, θ_min_ = 3.1°
ω/2θ scans	*h* = −8→8
Absorption correction: ψ scan (North *et al.*, 1968)	*k* = 0→8
*T*_min_ = 0.954, *T*_max_ = 0.977	*l* = 0→14
1123 measured reflections	3 standard reflections every 200 reflections
1123 independent reflections	intensity decay: 9%

### Refinement

**Table:**

Refinement on *F*^2^	Primary atom site location: structure-invariant direct methods
Least-squares matrix: full	Secondary atom site location: difference Fourier map
*R*[*F*^2^ > 2σ(*F*^2^)] = 0.058	Hydrogen site location: inferred from neighbouring sites
*wR*(*F*^2^) = 0.173	H-atom parameters constrained
*S* = 1.00	*w* = 1/[σ^2^(*F*_o_^2^) + (0.1*P*)^2^ + 0.23*P*] where *P* = (*F*_o_^2^ + 2*F*_c_^2^)/3
1123 reflections	(Δ/σ)_max_ = 0.001
82 parameters	Δρ_max_ = 0.26 e Å^−^^3^
0 restraints	Δρ_min_ = −0.24 e Å^−^^3^

### Special details

**Table:**

Geometry. All e.s.d.'s (except the e.s.d. in the dihedral angle between two l.s. planes) are estimated using the full covariance matrix. The cell e.s.d.'s are taken into account individually in the estimation of e.s.d.'s in distances, angles and torsion angles; correlations between e.s.d.'s in cell parameters are only used when they are defined by crystal symmetry. An approximate (isotropic) treatment of cell e.s.d.'s is used for estimating e.s.d.'s involving l.s. planes.
Refinement. Refinement of *F*^2^ against ALL reflections. The weighted *R*-factor *wR* and goodness of fit *S* are based on *F*^2^, conventional *R*-factors *R* are based on *F*, with *F* set to zero for negative *F*^2^. The threshold expression of *F*^2^ > σ(*F*^2^) is used only for calculating *R*-factors(gt) *etc*. and is not relevant to the choice of reflections for refinement. *R*-factors based on *F*^2^ are statistically about twice as large as those based on *F*, and *R*- factors based on ALL data will be even larger.

### Fractional atomic coordinates and isotropic or equivalent isotropic displacement parameters (Å^2^)

**Table:**

	*x*	*y*	*z*	*U*_iso_*/*U*_eq_	
O1	1.1983 (3)	0.2968 (2)	−0.07350 (16)	0.0520 (6)	
O2	1.3969 (3)	0.5931 (3)	−0.13385 (19)	0.0636 (7)	
O3	1.6159 (2)	0.3997 (3)	−0.17937 (17)	0.0531 (6)	
C1	0.8429 (3)	0.0447 (3)	0.0519 (2)	0.0431 (7)	
H1A	0.7382	0.0734	0.0859	0.052*	
C2	0.9507 (3)	0.1863 (4)	0.0139 (2)	0.0444 (7)	
H2A	0.9186	0.3114	0.0235	0.053*	
C3	1.1039 (3)	0.1452 (4)	−0.0376 (2)	0.0425 (7)	
C4	1.3668 (3)	0.2569 (4)	−0.1199 (2)	0.0460 (7)	
H4A	1.3449	0.1844	−0.1896	0.055*	
H4B	1.4456	0.1845	−0.0658	0.055*	
C5	1.4535 (3)	0.4417 (4)	−0.1439 (2)	0.0441 (7)	
C6	1.7243 (4)	0.5536 (4)	−0.2101 (3)	0.0617 (8)	
H6A	1.8350	0.5067	−0.2354	0.093*	
H6B	1.7513	0.6340	−0.1452	0.093*	
H6C	1.6595	0.6240	−0.2705	0.093*	

### Atomic displacement parameters (Å^2^)

**Table:**

	*U*^11^	*U*^22^	*U*^33^	*U*^12^	*U*^13^	*U*^23^
O1	0.0553 (11)	0.0450 (11)	0.0577 (12)	0.0023 (9)	0.0163 (9)	0.0020 (9)
O2	0.0667 (14)	0.0491 (12)	0.0760 (16)	0.0059 (10)	0.0130 (11)	−0.0036 (11)
O3	0.0517 (11)	0.0536 (12)	0.0555 (12)	−0.0040 (9)	0.0125 (9)	−0.0034 (9)
C1	0.0441 (14)	0.0464 (14)	0.0381 (14)	0.0046 (11)	−0.0002 (10)	−0.0050 (11)
C2	0.0433 (14)	0.0448 (14)	0.0444 (16)	0.0021 (11)	0.0006 (11)	−0.0049 (12)
C3	0.0451 (14)	0.0459 (14)	0.0356 (14)	0.0001 (11)	−0.0011 (11)	0.0007 (11)
C4	0.0395 (13)	0.0500 (15)	0.0473 (15)	−0.0007 (11)	−0.0018 (11)	0.0007 (12)
C5	0.0519 (15)	0.0476 (15)	0.0314 (13)	0.0028 (12)	−0.0031 (11)	−0.0023 (11)
C6	0.0703 (19)	0.0603 (18)	0.0553 (18)	−0.0192 (15)	0.0100 (14)	0.0034 (15)

### Geometric parameters (Å, °)

**Table:**

O1—C3	1.372 (3)	C2—C3	1.370 (4)
O1—C4	1.440 (3)	C2—H2A	0.9300
O2—C5	1.164 (3)	C4—C5	1.499 (4)
O3—C5	1.347 (3)	C4—H4A	0.9700
O3—C6	1.424 (3)	C4—H4B	0.9700
C1—C2	1.385 (4)	C6—H6A	0.9600
C1—C3^i^	1.419 (3)	C6—H6B	0.9600
C1—H1A	0.9300	C6—H6C	0.9600
			
C3—O1—C4	116.7 (2)	C5—C4—H4A	110.2
C5—O3—C6	116.9 (2)	O1—C4—H4B	110.2
C2—C1—C3^i^	118.4 (2)	C5—C4—H4B	110.2
C2—C1—H1A	120.8	H4A—C4—H4B	108.5
C3^i^—C1—H1A	120.8	O2—C5—O3	125.3 (3)
C3—C2—C1	121.2 (2)	O2—C5—C4	128.6 (3)
C3—C2—H2A	119.4	O3—C5—C4	106.1 (2)
C1—C2—H2A	119.4	O3—C6—H6A	109.5
C2—C3—O1	116.0 (2)	O3—C6—H6B	109.5
C2—C3—C1^i^	120.4 (2)	H6A—C6—H6B	109.5
O1—C3—C1^i^	123.5 (2)	O3—C6—H6C	109.5
O1—C4—C5	107.6 (2)	H6A—C6—H6C	109.5
O1—C4—H4A	110.2	H6B—C6—H6C	109.5
			
C3^i^—C1—C2—C3	0.9 (4)	C3—O1—C4—C5	−175.2 (2)
C1—C2—C3—O1	178.9 (2)	C6—O3—C5—O2	−1.9 (4)
C1—C2—C3—C1^i^	−0.9 (4)	C6—O3—C5—C4	178.7 (2)
C4—O1—C3—C2	175.4 (2)	O1—C4—C5—O2	−3.6 (4)
C4—O1—C3—C1^i^	−4.8 (4)	O1—C4—C5—O3	175.74 (19)

Symmetry codes: (i) −*x*+2, −*y*, −*z*.

### Hydrogen-bond geometry (Å, °)

**Table:**

*D*—H···*A*	*D*—H	H···*A*	*D*···*A*	*D*—H···*A*
C6—H6C···Cg1^ii^	0.97	2.98	3.674 (2)	130

Symmetry codes: (ii) −*x*+3/2, *y*−1/2, −*z*+1/2.
